# Factors Determining Women’s Attitudes and Knowledge Toward Breast Cancer Screening: A Systematic Review

**DOI:** 10.3390/healthcare13131605

**Published:** 2025-07-04

**Authors:** Dimitra Georga, Afroditi Zartaloudi, Maria Saridi, Evangelos C. Fradelos, Erasmia Rouka, Pavlos Sarafis, Dimos Mastrogiannis, Aikaterini Toska

**Affiliations:** 1 Department of Nursing, University of Thessaly, 415 00 Larissa, Greece; dimig17@yahoo.gr (D.G.); msaridi@uth.gr (M.S.); efradelos@uth.gr (E.C.F.); errouka@uth.gr (E.R.); psarafis@gmail.com (P.S.); 2 Department of Nursing, University of West Attica, 122 43 Egaleo, Greece; mastrogiannisds@gmail.com

**Keywords:** breast cancer, screening, attitudes, life satisfaction, general attitudes toward life

## Abstract

**Background/Objectives**: Breast cancer (BCA) is one of the most common cancers affecting women worldwide. Screening has been linked to up to a 33% reduction in breast cancer-related deaths by helping detect tumors at an early stage. The successful implementation of community-based breast cancer screening programs depends on understanding the attitudes of women within the target community. This study aims to systematically review the literature to assess the association between women’s attitudes toward breast cancer screening and their life satisfaction and general attitudes toward life. We also aimed to examine women’s attitudes toward breast cancer screening recommendations and the factors that influence these attitudes. **Methods**: A systematic review of English-language literature was carried out. PubMed and Scopus were searched up to November 2024 for studies that met the predefined inclusion criteria. Methodologic quality was assessed using the adapted Newcastle–Ottawa Scale for cross-sectional studies and the corresponding scale for cohort studies. **Results**: Eleven studies met the inclusion criteria. The percentage of women who had undergone at least one breast cancer screening ranged from 8.3% to 94.5%. Women’s attitudes toward and participation in breast cancer screening were linked to psychological, social, and demographic factors. Lower levels of life satisfaction, self-efficacy, and perceived control or mastery in life, along with higher levels of non-work-related stress, and higher levels of optimism, were associated with a lower likelihood of participating in breast cancer screening.

## 1. Introduction

Breast cancer (BCA) is the most common cancer affecting women worldwide [1]. According to the International Agency for Research on Cancer (IARC), a branch of the World Health Organization (WHO), breast cancer surpassed lung cancer as the most frequently diagnosed cancer in 2020, accounting for 11.7% of all cancer cases [2]. Furthermore, the latest GLOBOCAN 2022 data report that breast cancer is responsible for approximately 2.3 million new cases and 665,000 deaths globally [3].

Breast cancer screening has been linked to up to a 33% reduction in breast cancer-related mortality by enabling the detection of early-stage tumors, which typically have a more favorable prognosis [4].

In response to this, the European Council published a new recommendation (2022/C 473/01) on 9 December 2022 titled “Council recommendation on strengthening prevention through early detection: A new EU approach on cancer screening” which replaces Council Recommendation 2003/878/EC. This recommendation aims to promote cancer screening along the entire cancer care pathway as part of a new cancer prevention strategy for the EU [5].

Moreover, many Western countries have implemented mammography (MMG) screening programs aimed at reducing breast cancer deaths [6]. Evidence-based guidelines recommend universal screening with mammography for breast cancer [4]. However, despite their proven importance, participation rates in screening programs vary widely and remain significantly low [7]. For example, Lau et al. (2022) report findings from a recent study on screening monitoring in 17 European Union countries, which showed that nearly half failed to reach the 70% coverage threshold for breast and cervical cancer screening, as recommended by the European Council [8].

The problem is even more serious in developing countries, where the level of utilization/uptake remains low [9] despite higher disease burdens [10,11], making the understanding of the factors affecting participation in breast cancer screening essential for designing effective interventions.

Both systemic and individual factors must be taken into account in order to understand these variations. Breast cancer screening is considered a health-promoting behavior, influenced by complex factors [12]. Understanding these factors can contribute to the design of appropriate and targeted interventions to increase women’s participation in screening. In addition, successful implementation of community-based breast cancer screening programs requires an understanding of the attitudes of women in the target community. The success of such programs depends primarily on screening uptake and a high level of women’s participation in them. Therefore, assessing factors related to women’s attitudes within the community can help identify and remove barriers to screening [13].

One such factor is the variation in screening guidelines across countries. Recommendations regarding cancer screening vary from country to country and may also differ even between organizations within the same country [14]. A 2018 report that looked at cancer screening guidelines across 21 high-resource countries found that, when it comes to breast cancer, the recommendations were generally quite similar. Most countries, particularly in Europe, advised women to begin screening between the ages of 50 and 69, with the exception of Japan, where the screening interval was not specified. According to the same report, the American College of Radiology had the widest age range for screening and was the only guideline in the world that recommended an annual screening interval, while, in contrast, the United Kingdom had the longest interval (every three years) [14].

Additionally, previous studies have identified various socioeconomic and demographic factors, such as race and ethnicity [15,16], educational level [17,18,19], age [19], and financial constraints [18,20] as key barriers to compliance with breast cancer screening. On the other hand, few empirical studies have investigated the relationship between breast cancer screening behaviors and psychological factors, such as life satisfaction [21,22,23].

Life satisfaction is a reliable dimension of quality of life, encompassing physical, mental, and social well-being [24]. Life satisfaction is crucial for people’s health [24,25]. Better life satisfaction is associated with improved health outcomes [26], lower overall mortality rates [27], and health-promoting behaviors such as physical activity, less smoking, and a healthier diet [28]. It has also been shown that women who are satisfied with their lives are likely to take better care of their health compared to those whose life satisfaction is low [29].

Although the importance of psychological factors, such as social isolation, emotional support, and life satisfaction, in influencing decisions about breast cancer screening is becoming more widely acknowledged, no systematic reviews have yet summarized these associations.

The present study aims to systematically review the literature to assess the degree of the association between women’s attitudes toward breast cancer screening and their life satisfaction and general attitude towards life. The sub-objectives are to investigate the degree of women’s compliance with breast cancer screening, as well as the factors associated with these attitudes.

## 2. Materials and Methods

This systematic review was guided by the PICO framework to define the study objectives [30,31] (Table 1), and data collection was carried out following the PRISMA 2020 guidelines [32]. The literature search was conducted in November 2024 using the PubMed and Scopus bibliographic databases.

Each database was systematically searched using keywords related to women’s attitudes toward breast cancer screening, life satisfaction, and general outlook on life. Specifically, the following keywords were used: breast carcinoma, breast cancer, breast neoplasm, breast malignancy, BCA, self-examination, mammography, MMG, prevention, screening, screening participation, screening hesitancy, screening attitudes, preventive health behavior, attitudes towards screening, screening practice, screening behavior, screening uptake, screening adherence, screening practice, screening use, life satisfaction, satisfaction with life, gratification, subjective well-being, general attitude towards life, attitude toward life, psychological factors, optimism, easygoing, introversion, exoversion, emotional expression, worldview, meaning of life.

The above keywords were combined using the Boolean operators AND and OR.

In the PubMed bibliographic database, specific filters were applied to refine the search results. These included text availability (Free full text), language of publication (English), participant gender (Female), and age group (Adult: 19+ years).

In the Scopus database, restrictions were also applied. These included limiting the document type to articles, the language to English, and the source type to journals.

In contrast, no restrictions were applied regarding the year of publication in either of the bibliographic databases.

The search was conducted within the title and abstract fields of the publications.

Two independent reviewers conducted the literature search and data extraction. Any conflicts between them were resolved through consensus or by consulting a third author.

### Inclusion and Exclusion Criteria

The inclusion criteria for this study were as follows: studies published in English in peer-reviewed journals, regardless of the year of publication, conducted in any country, and employing a quantitative research design.

In contrast, the exclusion criteria comprised studies published in languages other than English; studies that focused on general cancer screening rather than specifically on breast cancer screening; studies reporting on breast cancer in men; and studies that presented data only for a specific subgroup of the eligible population—such as immigrants or women from urban or rural areas—except age-specific groups. Usually, breast cancer screening programs for the general population do not include women under 40 years old. This contributes to diagnostic delays. The rate of breast cancer growth in younger women is significantly higher compared to that in older women. Increasing awareness and educational programs to help recognize signs and symptoms in young women, and also the development of clear diagnostic guidelines and screening strategies have been recommended. Therefore, the inclusion of studies involving younger women offers valuable information regarding the attitudes and awareness of this underserved population as well [33].

Additionally, studies involving women who had already been diagnosed with breast cancer or were breast cancer survivors were excluded. Non-original research articles, including dissertations, reviews, case reports, oral and poster presentations, and book chapters, were also excluded. Additionally, we did not include studies that used a qualitative research design. While combining qualitative and quantitative data could provide useful insights, there are methodological obstacles to overcome. As Sandelowski et al. (2006) [34] point out, resolving the methodological differences between study types is necessary for a mixed research synthesis. This can make synthesis within a single analytical framework more difficult [34]. Therefore, in order to ensure the consistency and comparability of results, we only included quantitative studies in this review.

**Figure 1:**
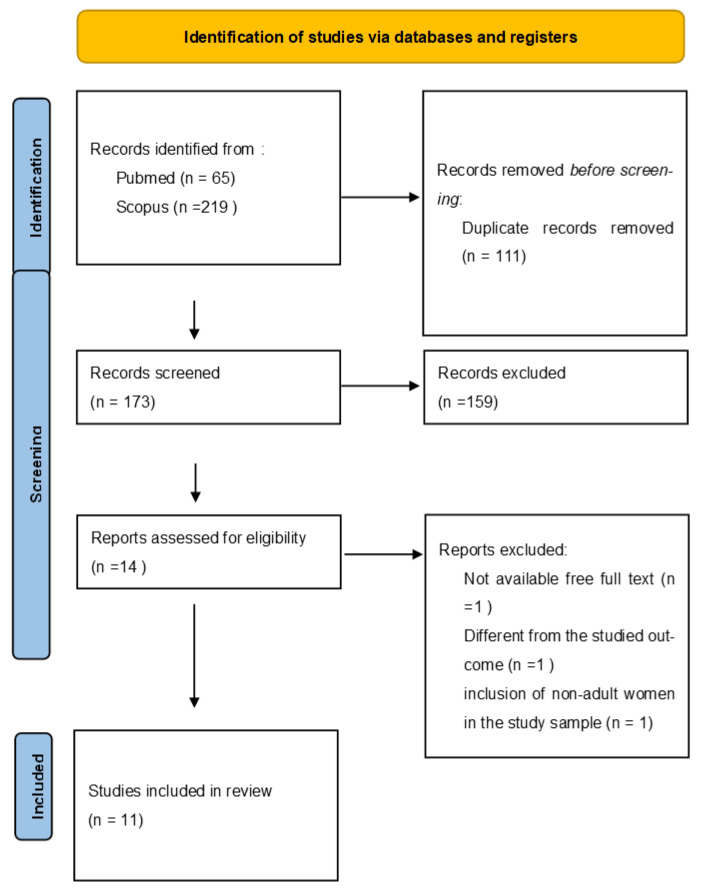
Flow chart of the identification of studies via databases and registers.

Finally, systematic reviews, meta-analyses, pilot studies, and descriptive reviews, were not considered for inclusion (see Figure 1).

## 3. Results

The initial search, with the restrictions that had been set, yielded 284 results, of which 111 were removed due to multiple entries/appearances in the results before reading the title and abstract. Subsequently, after reading the title and abstract, 159 entries were excluded for not meeting the study entry criteria or due to a topic not relevant to the issue under study. Of the 14 articles that remained, 3 were excluded after reading the full text, one due to the full text not being freely available, one due to a different study outcome, one due to the inclusion of non-adult women in the study sample (participants from 15 years of age) (flow diagram).

Of the eleven studies included in this review, eight were conducted in developed countries [23,35,36,37,38,39,40,41] and three in developing countries [42,43,44], according to the IMF’s World Economic Outlook classification (last updated April 2023) [45]. Specifically, three studies were conducted in the United States [39,40,41], one in Canada [35], one in Israel [23], one in Japan [36], one in Norway [37], one in Sweden [38], one in China [43], one in Lebanon [42], and one in Malaysia [44] (Table 2 and Table 3).

Of the included studies, nine addressed breast cancer screening [23,35,37,38,39,40,41,42,44] and two addressed screening for both breast and uterine cancer [36,43].

The age composition of participants among the different studies varies, with some studies including women of any age over 18 years [37], and other including women of specific age groups, such as those aged 50 to 74 years [23,35], 60 years and older [39,44], 21 to 77 years [36], 40 years and older [41], up to 70 years [43], or even up to 75 years [42]. Similarly, the percentages of women who have undergone at least one breast cancer screening range from 8.3% [44] to 94.5% [35].

Regarding the factors that were associated with women’s attitudes toward and participation in breast cancer screening across the various studies, a lower probability of participation or intention to participate in screening was linked to the existence of mental health problems [23], the absence or limited levels of emotional support received [23], low social participation [38] and the feeling of social isolation [23], lack of belief in the effectiveness of mammography in reducing the threat of cancer [37,39,40], not regularly undergoing mammography screening [37] or annual medical check-ups [39], higher levels of predisposition to anxiety [37], the absence of experience in the close friendly or family environment of a person diagnosed with breast cancer [35,39,44], the lack of discussion regarding screening with the healthcare provider and the lack of recommendation from them [35,39], the absence of encouragement from the spouse [42] as well as low perceived sensitivity to the occurrence of breast cancer [39,42].

Sociodemographic factors such as belonging to racial/ethnic minorities [41], lower socioeconomic status [41,42], having a disability [41], lack of insurance [40,41], lower educational level [36,39,42], and lower family income [36] were also associated with a lower likelihood of participating in breast cancer screening. Older age has been associated in some studies with a lower likelihood of participating in breast cancer screening [36,39,43], and in others with a higher likelihood [35,37,41,42]. Marital status has also been associated with a higher likelihood of participating in breast cancer screening [36,39,42].

Regarding the relationship between life satisfaction and general attitudes toward breast cancer screening, the studies included in this systematic review showed that lower levels of life satisfaction are associated with a lower likelihood of participating in breast cancer screening [23]. Lower levels of self-efficacy [40], a low sense of control or mastery in life, and higher levels of non-work-related stress [38], as well as higher levels of optimism [41] were also associated with a lower likelihood of participating in breast cancer screening.

## 4. Assessment of the Methodological Quality of the Included Studies

The assessment of the methodological quality of the studies was performed using the adapted Newcastle–Ottawa Scale for cross-sectional studies [46] and the corresponding scale for cohort studies [47]. A score of seven stars or more on either scale indicates a low risk of bias, while a score of six stars or fewer indicates a high risk of bias [48].

**Figure 2:**
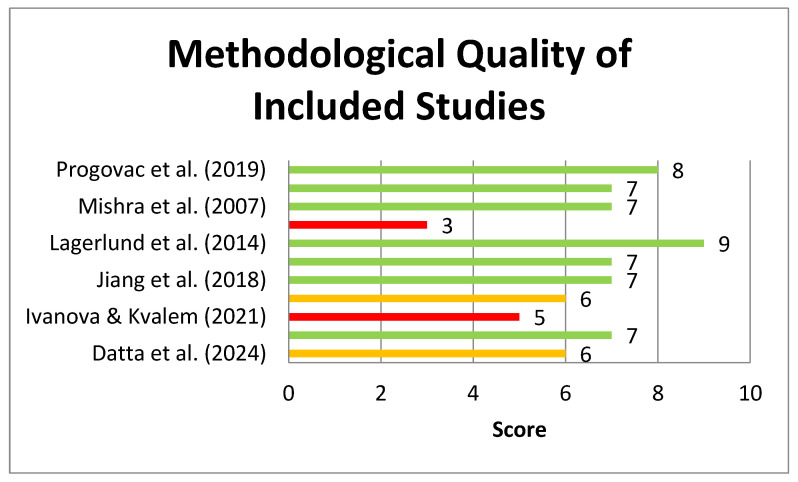
Methodological quality of the included studies [23,37,38,40,41,43].

Most of the studies included in the review were assessed as having a low risk of bias (Figure 2). Regarding sampling, although the majority of studies included representative samples of the target population, few provided sufficient documentation or information on sample size calculation. Additionally, only a limited number of studies reported details about non-participants and their characteristics.

Almost all of the included studies relied on subjective measures, primarily using self-reported data from participants, which introduces the potential for information bias [49].

Almost all of the studies also employed statistical methods to control for confounding factors, which contributes to strengthening the validity of their results. In addition, appropriate statistical tests were used across all studies, with clear presentation of the tests applied and the relationships between the measured variables.

## 5. Discussion

Τhe present study aimed to systematically review the literature to identify factors that inhibit or promote women’s participation in breast cancer screening, as well as to investigate the relationship between these attitudes, life satisfaction, and general attitude toward life.

Breast cancer screening is crucial for early diagnosis and treatment [50,51]. However, despite its importance and the support of both regional and international authorities, many countries have yet to achieve optimal screening coverage rates in the general population [8].

In the studies included, the percentage of women who had undergone at least one breast cancer screening ranged from 8.3% to 94.5%. The differences in national guidelines may help explain this wide variation in screening participation observed in the present systematic review. The differences could also be due to the different age compositions of participants in the studies included in this systematic review, as well as the fact that some studies were conducted in developing countries. Moreover, the observed variation may reflect differences in women’s attitudes toward breast cancer screening, as prior research has shown that positive attitudes are strongly associated with higher participation rates [52].

Our findings show that women’s attitudes toward breast cancer screening recommendations play a crucial role in participation decisions. Specifically we found that women who had discussed screening with a healthcare provider [35] and received an official invitation for screening [35] were significantly more likely to participate. On the other hand, a lack of recommendation from healthcare professionals [39] was associated with a lower likelihood of screening. Moreover, the belief in mammography’s effectiveness in reducing cancer risk was positively associated with screening intention [37]. These findings indicate that understanding of screening recommendations and exposure to such recommendations are linked to women’s willingness to follow them.

From the present systematic literature review, we also found that mental health problems are among the key factors negatively associated with breast cancer screening participation. A number of studies, both on breast cancer [53,54,55,56] and on other types of cancer [57,58,59] have shown that people with mental illness are less likely to take part in screening programs. Furthermore, it has been shown that the participation rates vary depending on the type and severity of the mental illness [60]. This knowledge demonstrates the need to design and implement appropriate and targeted interventions for the population of women with mental illnesses to increase their participation in breast cancer screening.

In addition to the above, the relationships between social support, positive health outcomes, and well-being are well established, and individuals who have social and community ties have been shown to have lower rates of morbidity and mortality compared to those who lack social support [61]. In contrast, social isolation has been identified as an independent major risk factor for all-cause mortality [62]. Social support has also been shown to promote healthy behaviors and, together with social relationships, influence screening behaviors [63]. The above is in agreement with our findings, where the absence or limited levels of emotional support received, low social participation, and the feelings of social isolation emerged as factors associated with reduced participation in breast cancer screening.

The reasons for the potentially protective effects of social support on women’s cancer screening compliance are unclear. First of all, it has been suggested that social support could act as a moderator and may help reduce the negative impact of stressful events. Social support may help relieve anxiety related to cancer screening, as well as provide the necessary resources that encourage screening behaviors. Furthermore, according to the “main effect” model, social support may influence health and health behaviors through a more direct pathway. Social relationships may directly influence screening behavior through social norms or peer pressure. In addition to the main effect, others have hypothesized that social support could influence cancer screening by increasing knowledge, which, in turn, may increase screening by providing women with a more accurate personal perception of risk and helping them overcome barriers to screening. Finally, it has been suggested that the health and healthcare habits of individuals with lower socioeconomic status may benefit more from social support, as it may be directly related to helping them overcome financial and educational barriers, as well as increasing access to social capital [63].

In some cases, familiarity with and knowledge of a previous experience of cancer involving family members or other loved ones can trigger negative emotions and memories that may impact one’s life and health-related decisions. Conversely, individuals who do not have experience with friends or relatives affected by cancer may respond differently to a screening invitation, as they may not perceive themselves to be as susceptible to the condition [64]. Among the studies included in this systematic review, the absence of experience in the close friendship or family environment of a person diagnosed with breast cancer was negatively associated with women’s screening behaviors.

Related to the above is the concept of perceived risk, which plays a central role in many theories of health behavior, including the Health Belief Model, Protection Motivation Theory, and the Theory of Reasoned Action/Theory of Planned Behavior [65]. In the present study, two of the included studies found that women who perceived themselves as less susceptible to breast cancer were also less likely to participate in screening. It is important to recognize that the term “risk” has different meanings for different people, including experts and the public. Research into the perceived risk of breast cancer has shown that laywomen have a different set of beliefs about the causes, curability, and risk factors of breast cancer compared to those of health professionals. Understanding how women perceive their own risk of breast cancer gives us valuable insight into how they interpret health messages and make decisions about screening. This knowledge facilitates the development of effective interventions to improve risk communication [66].

Patients’ cancer screening is significantly influenced by healthcare providers [67], who can serve as a key source of health information by evaluating their eligibility for screening, discussing a course of action, and helping to coordinate screening tests and follow-up care [68]. Moreover, one of the biggest barriers to screening, particularly for women, is low health literacy, which limits individuals’ ability to acquire, process, and understand information about cancer screening methods and symptoms. This, in turn, can significantly affect both diagnosis and treatment decisions [69]. It has also been shown that the public has limited knowledge about the way screening programs affect them and that only 1.5% of Europeans are aware of the benefits of participating in breast cancer screening [70]. Similarly, in three of the studies included in this systematic review, women’s lack of belief in the effectiveness of mammography to reduce cancer risk was negatively associated with their attitudes toward screening.

Health professionals play a significant role in helping women decide on cancer screening. By discussing the possible advantages and drawbacks of screening, they can help them make informed decisions that match their values and preferences. Healthcare providers play an important role in the control of these cancers by advising women to seek medical care for unusual symptoms. In addition, they can affect their screening behaviors and increase their confidence in screening by helping them understand the importance of regular screening [67]. Counseling focused on patients’ basic concerns has been proven more successful than untailored discussions about behavioral components [71]. This was also found in the studies included in the present systematic review, which revealed the negative impact of not discussing breast cancer screening with a healthcare provider and the lack of a recommendation.

Sociocultural beliefs, values, and norms have been shown to hinder women’s access to breast cancer screening services [72]. For example, some traditional Hispanics believe that only a husband or lover should touch a woman’s breasts. Therefore, spousal permission may be required to enhance breast cancer screening in this population [73]. Similarly, in several countries in the Middle East and the Arab world, the culture is conservative, and men typically manage many of women’s decisions, choices, and actions [74]. In this regard, spouses can play an influential role as part of cultural subjective norms, because the adoption or non-adoption of breast health-seeking behaviors depends on the locus of control of women’s health [75]. This finding was also highlighted in the present systematic review by a Lebanese study [42] where one of the factors inhibiting women’s participation in breast cancer screening was the lack of encouragement from the spouse. These results are important for improving breast cancer screening rates among women belonging to ethnic minorities, highlighting the need for a culturally appropriate approach [73].

Sociodemographic factors such as racial/ethnic minority status were also linked to lower rates of breast cancer screening. It has been shown that women from racial, ethnic, and cultural minority groups are less likely to follow breast cancer screening guidelines, which can lead to delayed diagnosis, poorer prognosis, and higher mortality rates. This is influenced by potential disparities or barriers that these women face when trying to access screening services [76]. One study found that pain and embarrassment linked with mammography, low income and lack of health insurance, insufficient knowledge about breast cancer screening, lack of a doctor’s recommendation, lack of trust in hospitals and doctors, language barriers, and lack of transportation were the most frequently identified factors influencing minority women’s participation in screening [77].

Other socioeconomic factors that negatively affected women’s participation in breast cancer screening, as identified in this systematic review, were lower socioeconomic status, lack of insurance, and lower educational level. Women with lower socioeconomic status have less access to health promotion resources, which could affect their participation in health promotion programs like breast cancer screening [78]. It has also been shown that low-income women do not receive enough information about screening. As a result, they may not respond to invitations because they do not understand what the screening involves or are not aware of the disease’s risk factors, signs, or symptoms [79]. Poverty also plays a major role, as women with lower economic status may have to prioritize providing for their families over participating in preventive care behaviors [80]. Regarding health insurance, it offers financial protection against the multiple costs that may arise from participating in a screening program or, more significantly, from the diagnosis of a suspicious breast neoplasm [79]. Finally, in terms of educational level, education plays a key role in shaping beliefs and reducing unfavorable attitudes toward interventions aimed at enhancing knowledge about health and disease [81].

The existence of disability was also reported in this study as one of the factors negatively associated with women’s participation in breast cancer screening. People with disabilities face several barriers to accessing screening, including lack of accessibility (information, transportation, equipment, and facilities), limited financial capacity, communication difficulties, and negative attitudes from health professionals. They are also, on average, poorer and have lower levels of education, two known predictors of low participation in screening, as demonstrated by our study too [82].

Regarding age, the results of the studies included in this review were conflicting, with older age being associated in some studies with a lower likelihood of participation in screening, and in others with a higher likelihood.

Finally, in the present study, “married” marital status was associated with a greater likelihood of participating in breast cancer screening. Marriage may promote healthy behaviors for at least two reasons. Specifically, the health behaviors of married women are often monitored and influenced by their spouses, and marriage may also provide emotional support for maintaining healthy behaviors [83]. Additionally, due to the influence of this group on women’s health-seeking behaviors, interventions aimed at increasing screening intention should particularly target spouses. Therefore, education and awareness about breast cancer should be provided to those with influence to help women participate in screening [84].

Studies included in this systematic review also showed that lower levels of life satisfaction are associated with a lower likelihood of participating in breast cancer screening [23]. Women who are satisfied with their lives are more likely to take better care of their health than those who are less satisfied. Research shows that younger women, those with higher education, and those who are married tend to report higher life satisfaction, enjoying their family life and professional careers, and are more likely to be in good health [29].

The concept of optimistic bias is also an important factor in disease prevention. It refers to the psychological tendency people have to believe they are less likely than others to experience negative future events, and more likely to experience positive future events. It generally shows the tendency of individuals to underestimate the chances of experiencing bad events or diseases and to overestimate the chances of experiencing positive events. When individuals have an optimistic bias, they tend to make more optimistic judgments about their own situations than about the situations of others [85]. Various explanations have been put forward for this phenomenon. According to one of them, optimistic bias reduces the anxiety that would be caused by acknowledging personal vulnerability. According to this interpretation, optimistic bias increases when a disease is considered serious. Another hypothesis suggests that people judge their own susceptibility to diseases as lower than that of others in order to boost self-esteem. Self-esteem is threatened when a risk is not avoided, especially if it is avoidable. A third theory describes optimistic bias as the result of a cognitive error. Rare diseases, for example, lead to optimistic bias, as people fail to consider that the same conditions (i.e., the rarity of the disease) apply to everyone. Finally, people tend to conclude the future based on past experiences, such as “I haven’t had cancer, so I won’t get it in the future” [86]. Optimistic bias can lead people to underestimate health-related risks and may influence how strictly patients adhere to treatment plans. Furthermore, optimistic bias has a significant impact on disease prevention [85]. This could potentially explain the finding of the present study that higher levels of optimism were associated with a lower likelihood of participating in breast cancer screening.

## 6. Limitations

This systematic review has some limitations. First, although a comprehensive search strategy was used, the exclusion of unpublished literature and the inclusion of only studies published in English may have resulted in not capturing all relevant literature. Additionally, this study did not investigate the possible effect of healthcare provider characteristics (e.g., education, age) on the outcomes of interest. Future research could explore these effects. Moreover, the age range of participants differed across studies, both developed and developing countries were included, and there were variations in the time intervals used to monitor women’s compliance with screening. Health systems also differ among the countries included in the review, and in some, such as the USA, participation in screening may require payment. Therefore, the results may not be fully comparable.

## 7. Conclusions

Breast cancer screening, when used appropriately, is associated with reduced mortality, improved population health, and lower healthcare costs. Increased participation of women in screening programs is essential for their success. Understanding the factors that influence women’s attitudes and behaviors toward breast cancer screening—just as with any other screening program—is crucial for increasing participation through targeted and appropriate interventions.

This systematic review emphasizes how important psychological factors are in influencing women’s attitudes and behaviors about breast cancer screening, especially life satisfaction and overall outlook on life. Despite the identification of many structural and sociodemographic factors, the results highlight a strong correlation between reduced screening participation and worse life satisfaction. This implies that initiatives to raise screening rates should include tactics to improve women’s psychological health, in addition to addressing structural obstacles. Public health programs can become more comprehensive and effective by addressing this underexplored field. This connection should be further explored in future studies to help inform practice and policy.

## Figures and Tables

**Table 1:** Analysis of the application of the PICO methodology in the systematic literature review.

Population	Women
Intervention (Influential Factors)	General Attitude Toward Life, Life Satisfaction, and Quality of Life
Comparison	Women who undergo breast cancer screening compared to those who do not
Outcomes	Breast cancer screening

**Table 2:** Association between life satisfaction and attitudes toward life and breast cancer screening.

Authors and Year of Publication	Relationship Between Life Satisfaction and Attitudes Toward Life and Women’s Attitudes Towards Breast Cancer Screening
Datta et al. (2024) [23]	Lower levels of life satisfaction were associated with a lower likelihood of participating in breast cancer screening.
Bawalle et al. (2024) [36]	Happiness levels, myopic view of the future, anxiety about later life, and perception of health status were not significantly associated with participation in breast cancer screening.
Oι Progovac et al. (2019) [41]	Higher levels of optimism were associated with a lower likelihood of participating in breast cancer screening.
Lagerlund et al. (2014) [38]	A lower likelihood of participating in breast cancer screening was associated with a low sense of control or mastery over life and higher levels of non-work-related stress.
Rahmah et al. (2013) [44]	A greater likelihood of undergoing screening mammography was associated with perceived general psychological state as “sometimes happy” compared to “happy” with their lives.
Mishra et al. (2007) [40]	Higher levels of self-efficacy were associated with a greater likelihood of participating in mammography screening.

**Table 3:** Women’s participation rate in breast cancer screening and its associated sociodemographic and psychological factors.

Authors and Year of Publication	Type of Study and Purpose	Number of Participants	Country of Study	Breast Cancer Screening Participation Rate	Sociodemographic and Psychological Determinants of Participation
Datta et al. (2024) [23]	Study Type: Cross-sectional study Aim: To assess the relationship between life satisfaction, emotional support, and social isolation and women’s compliance with the USPSTF breast cancer screening recommendations.	71,583 women aged 50–74 years	Israel	A total of 84% of women followed the recommended breast cancer screening guidelines.	A lower likelihood of participation in breast cancer screening was associated with the following: Experiencing mental health problems. Lack of or infrequent emotional support. Feelings of social isolation.
Bawalle et al. (2024) [36]	Study Type: Cross-sectional study Aim: To assess the relationship between financial literacy, financial education, and participation in breast and cervical cancer screenings.	1729 women aged 21–77 years	Japan	The breast cancer screening participation rate among Japanese women is 34%.	Breast cancer screening participation was positively associated with the following: Married marital status or being divorced. Higher educational level. Higher family income. The levels of health-related anxiety. Breast cancer screening participation was negatively associated with age and smoking behavior
Ivanova & Kvalem (2021) [37]	Study Type: Cross-sectional study Aim: To explore psychological determinants of mammography intention and defensive avoidance behaviors in breast cancer screening.	270 women aged 18 and older	Norway	In total, 66.5% of the participants reported having had at least one mammogram.	The intention to undergo mammography within the next two years was Positively associated with age, Negatively associated with trait anxiety, Positively associated with the belief in mammography’s effectiveness in reducing cancer risk, Positively associated with regular mammography (once a year or once every two years).
Oι Progovac et al. (2019) [41]	Study type: Longitudinal cohort study Aim: To investigate the association between psychological attitudes (optimism and cynical hostility) and the frequency of mammography screening, as well as the possible influence of sociodemographic factors on these relationships.	48,291 postmenopausal women 40 years and older	USA	A total of 13% of women had not undergone any mammography screening.	The following factors were linked to a greater likelihood of not undergoing preventive mammography screening: Older age. Belonging to racial/ethnic minorities. Lower socioeconomic status. Having a disability. Lack of insurance. Not having a regular doctor. Smoking habits, current or past. Less physical activity.
Abelson et al. (2018) [35]	Study Type: Cross-sectional study Aim: To investigate women’s attitudes toward and perspectives on screening mammography.	2000 women aged 50–74 years	Canada	A total of 5.5% of the participants had never undergone mammography.	A higher likelihood of undergoing mammography was associated with the following: Older age. Having a close friend or family member diagnosed with breast cancer. Receiving an invitation for screening. Discussing screening with a healthcare provider.
Jiang et al. (2018) [43]	Study Type: Cross-sectional study Aim: To investigate the relationships between breast and cervical cancer screening requirements and related health beliefs.	805 women aged 40–70 years	China	In total, 18.6% of participants had undergone breast cancer screening.	Breast cancer screening uptake was negatively associated with age but positively associated with educational level.
Elias et al. (2017) [42]	Study Type: Cross-sectional study Aim: To investigate patterns and determinants of mammography screening.	2400 women aged 40 to 75 years	Lebanon	In total, 4.4% of women had never heard of mammography. Of the 2295 women who had heard of mammography, 55% had never undergone a mammography screening.	The following were linked to a higher probability of undergoing mammography: Older age. Better socioeconomic status. Higher educational level. Married marital status. Higher perceived susceptibility to breast cancer. Ease of access to screening. Receiving encouragement from a spouse.
Lagerlund et al. (2014) [38]	Study Type: Prospective community-based cohort study Aim: To investigate women’s participation in a population-based breast cancer screening program.	11,409 women aged 44 to 72 years	Sweden	Out of the 69,746 women who were invited, 92% showed up for screening, while 8% did not.	The following were associated with a lower likelihood of participating in breast cancer screening: Not having a partner and living alone or only with children. Having one or three or more children compared to having two children. Low social participation
Rahmah et al. (2013) [44]	Study Type: Cross-sectional study Aim: To investigate the prevalence of mammography screening and the factors associated with its uptake among older women.	652 women aged 60 and older	Malaysia	Only 8.3% of women had ever had a mammogram in their life.	Women were more likely to have a mammogram if they had a family history of breast cancer.
Mishra et al. (2007) [40]	Study Type: Cross-sectional study Aim: To investigate the association between cultural and psychosocial factors with not receiving mammography.	809 women of Samoan origin aged 42 and older	USA	A total of 58.7% of participants had never had a mammogram.	∙ Lower likelihood of participating in breast cancer screening was associated with the following: Greater misconceptions about risk factors for the disease. Lack of insurance coverage. Placing less importance on the breast as a symbol of femininity. Disagreement about the effectiveness of mammography for detecting breast cancer in its early stages.
Michielutte et al. (1999) [39]	Study Type: Cross-sectional study Aim: To investigate psychosocial factors associated with breast cancer screening in older women.	719 women aged 60 and older	USA	A total of 88% of women had had a mammogram at some point in their lives. Among them, 50.4% had one in the past year, 29.8% between one and three years ago, and 7.8% more than three years ago.	The following factors were associated with a lower likelihood of screening with mammography in general or during the previous year: The absence of symptoms. Low perceived sensitivity to the disease. The feeling that the use of mammography is pointless. Lack of knowledge about mammography and where it is available. Lack of recommendation from a physician. Older age. Lower level of education. Single marital status. Lack of private insurance beyond public. Not having an annual medical check-up. No family history of breast cancer.

## Data Availability

Not applicable.
